# Challenges, Strategies, and Explanatory Mechanisms in Clinical Skills Remediation Programs in Undergraduate and Postgraduate Medical Education in Low- and Middle-Income Countries: Realist Review Protocol

**DOI:** 10.2196/89550

**Published:** 2026-06-02

**Authors:** Muhamad Reza Utama, Muhammad Anas, Yelvi Levani, Prattama Santoso Utomo, Rachmadya Nur Hidayah, Fithriyah Cholifatul Ummah, Cecilia Felicia Chandra, Yoyo Suhoyo, Ahmad Mochtar Jamil, Azhar Izha Fakhrussy

**Affiliations:** 1 Faculty of Medicine, Universitas Muhammadiyah Surabaya, Jl. Raya Sutorejo No.59, Dukuh Sutorejo, Surabaya, East Java, 60113, Indonesia, 62 08123141067; 2 Faculty of Medicine Public Health, and Nursing, Universitas Gadjah Mada, Yogyakarta, Special Region of Yogyakarta, Indonesia; 3 Faculty of Medicine, Universitas Airlangga, Surabaya, East Java, Indonesia

**Keywords:** realist review, medical student remediation, clinical learning environment, patient safety, physicians

## Abstract

**Background:**

Clinical skills deficits are a patient-safety concern, yet remediation remains underexamined, particularly in low- and middle-income countries, where faculty shortages, uneven access to simulation and supervised practice, inconsistent assessment, and stigma can hinder timely and effective support. The effects of trainee underperformance extend beyond the individual, with implications for patient care, supervisory workload, and team functioning. Although interest in remediation is increasing, the evidence base remains fragmented, dominated by high-income settings, and largely descriptive. A theory-driven review is therefore needed to explain how remediation works, for whom, and under what conditions.

**Objective:**

This realist review protocol aims to identify the main challenges and strategies reported in clinical skills remediation in undergraduate and postgraduate medical training, explain for whom these strategies work, in what contexts, and through which mechanisms, and develop an evidence-informed initial program theory for low- and middle-income country settings.

**Methods:**

Following the RAMESES (Realist and Meta-narrative Evidence Syntheses: Evolving Standards) for realist synthesis, this review will draw on a broad range of sources, including MEDLINE or PubMed, CINAHL, PsycINFO, ERIC, and Scopus, alongside gray literature such as dissertations, World Health Organization resources, medical education policy documents, and institutional reports. Sources published from January 2000 onward will be considered eligible, spanning empirical studies, program evaluations, and theory-informing documents focused on the remediation of clinical or procedural underperformance among medical learners at any training stage. Screening, extraction, and appraisal will be performed in duplicate. Extracted data will include learner characteristics, remediation triggers, intervention features, contextual conditions, candidate mechanisms, and outcomes related to competence, progression, and patient safety. Synthesis will apply retroductive reasoning to develop and refine context-mechanism-outcome configurations and a middle-range program theory.

**Results:**

Funding was secured through Badan Riset dan Inovasi Nasional (National Research and Innovation Agency of Indonesia; 2023). The protocol was registered in PROSPERO (International Prospective Register of Systematic Reviews) (CRD42023447029). Protocol development, stakeholder-informed scoping, preliminary literature familiarization, and initial program theory construction were completed by April 2026. Formal database and gray-literature searching is planned for mid-2026, followed by duplicate screening, relevance and rigor appraisal, data extraction, and iterative realist synthesis through 2026-2027. The primary review manuscript is targeted for submission in 2027.

**Conclusions:**

This review aims to move remediation scholarship beyond description, toward a clearer understanding of what works, for whom, and why. By bridging evidence, theory, and real-world practice, it hopes to guide the design of remediation systems that are contextually grounded, educationally sound, and ultimately capable of restoring safe clinical performance across both undergraduate and postgraduate medical training.

## Introduction

### The Patient-Safety Imperative of Clinical Skills Remediation

Clinicians who are skilled, safe, and operate efficiently in teams are an essential component of high-quality, safe health care. When physicians underperform, patients are placed at greater risk than necessary [1,2]. Performance problems can arise at any stage of the medical training trajectory—from preclinical undergraduate education to postgraduate residency and beyond—for reasons that include health and well-being, personal circumstances, workplace environment, and gaps in continuing professional development [3-5]. These problems are frequently complex and multifactorial, involving knowledge, technical skills, and professionalism domains [2,6-8].

Remediation is defined as the process by which a learner’s or practitioner’s underperformance is identified, addressed through structured intervention, and returned to a safe, defensible standard of practice [4,6]. More formally, it constitutes “an intervention, or series of interventions, required in response to an assessment of a threshold standard” [4,5,7,9-11]. Threshold standards are established by regulatory bodies to protect patients. Remediation interventions range from informal coaching and targeted retraining to structured, multicomponent programs involving reassessment, mentorship, and supervised practice [12-18].

Historically, remediation has been conceptualized primarily as a knowledge-correction exercise. However, this framing is insufficient; a practitioner’s performance is shaped by a complex array of contextual factors, including colleagues’ attributes, system resources, and organizational culture [19-23]. Viewing remediation as a purely educational exercise, divorced from contextual determinants of individual competence, is unlikely to address significant performance gaps.

An important conceptual shift has therefore occurred, reconceptualizing remediation as a process of behavioral change that requires understanding what is needed to produce lasting performance improvement for a given practitioner in a given context [24-26]. Recent scholars further frame successful remediation as a metacognitive reflective process, dependent on psychological safety, feedback credibility, guided reflection, and professional identity reconstruction [27-37]

### The Low- and Middle-Income Country Context and the COVID-19 Disruption

In low- and middle-income countries (LMICs), as classified by the World Bank fiscal year 2026 income classifications [38], clinical skills remediation faces additional and compounding challenges. These include limited faculty capacity for individualized support, variable access to simulation facilities and supervised clinical practice, uneven assessment infrastructures, and sociocultural meanings that may frame remediation as stigmatizing or punitive rather than developmental [22,39-42]. The COVID-19 pandemic markedly exacerbated these challenges. Medical schools across LMICs were forced to adopt online and hybrid learning delivery models, creating significant gaps in clinical skills acquisition and competence verification [6,43-48]. Unlike the high-income country (HIC) experience, LMIC institutions often lacked the infrastructure for robust remote clinical skills training, widening preexisting competence disparities [42,49-51]. Beyond its immediate effects, the pandemic-era shift toward remote and hybrid delivery models has underscored the longer-term need for remediation systems that can function across multiple delivery modalities, a consideration relevant well beyond the acute disruption period. Remediation programs must therefore be designed to function effectively in these evolving educational contexts. In Indonesia specifically—the largest LMIC context represented in this research team—national competency examination data suggest that clinical performance during internship is influenced by factors beyond summative assessment scores, underscoring the need for contextually sensitive, mechanism-informed approaches to remediation [35,43,52].

### Limitations of the Existing Evidence Base

The current evidence base is clinically useful but still contextually incomplete. The 2019 Best Evidence Medical Education review [53] and its 5-year update [54] assembled a substantial repertoire of remediation strategies for undergraduate and postgraduate learners, ultimately covering 121 interventions; however, most studies still focused primarily on knowledge and skills deficits, study quality remained variable, and many reports lacked explicit conceptual frameworks or robust attention to context, process, and implementation rather than outcomes alone.

The communication-skills review [55] reached similar conclusions from only 16 heterogeneous studies, most conducted in high-income settings and largely involving medical students—struggling learners were most often identified through Objective Structured Clinical Examination–type assessments, and remediation typically involved multimodal experiential practice with feedback, yet standardization, theoretical grounding, and outcomes-based evaluation remained limited.

Guideline synthesis by Chou et al [56] likewise framed remediation as a high-stakes, highly complex process involving learners, faculty, systems, and societal factors, while also highlighting persistent uncertainty about long-term outcomes, optimal duration and intensity, and the best ways to identify and support struggling learners early.

In the postgraduate literature, a scoping review by Cheong et al [57] of 101 articles showed that remediation is highly individualized and shaped by sociocultural, organizational, curricular, and hidden-curriculum influences; however, it also documented recurring barriers, including weak standardization, limited theoretical grounding, suboptimal screening and evaluation, faculty reluctance, and the time- and resource-intensive nature of remediation.

The Realist Synthesis of Doctor Remediation (RESTORE) realist review [58] moved the field forward by conceptualizing remediation as a behavior-change process rather than a simple educational correction. Across 141 studies, mostly from North America and the United Kingdom, it identified 29 context-mechanism-outcome (CMO) configurations showing how psychological safety, carefully framed feedback, destigmatization, perceived control, motivation, and guided reflection can foster insight and long-term practice change. Even so, RESTORE focused on practicing doctors rather than undergraduate and postgraduate trainees, and the wider remediation literature remains dominated by high-income settings.

Taken together, no existing review applies realist methodology specifically to clinical and procedural skills remediation for undergraduate and postgraduate trainees in LMIC contexts. Existing reviews tell us much more about what remediation programs contain than about how clinical skills remediation works for trainees in resource-constrained LMIC settings, where faculty scarcity, uneven assessment infrastructures, stronger power distance, and sociocultural stigma may substantially alter how mechanisms are triggered, negotiated, and maintained.

### Rationale for a Realist Synthesis

Realist synthesis is a theory-driven approach to evidence synthesis used to explain how and why complex interventions work differently across settings, rather than asking only whether they work on average [59-61]. Its central concern is the relationship between context, mechanism, and outcome—how particular conditions shape participants’ responses to an intervention and, in turn, influence whether desired, partial, or unintended outcomes occur [60]. This orientation is especially appropriate for clinical skills remediation, where improvement is unlikely to follow from intervention components alone. In practice, remediation outcomes are shaped by how educational supports interact with learner insight and motivation, the credibility of feedback, supervisory relationships, institutional culture, stigma, and available resources [62].

This makes realist synthesis a better fit than more design-restrictive review approaches for the current remediation literature, which is methodologically heterogeneous, unevenly theorized, and strongly shaped by context. Consistent with Realist and Meta-narrative Evidence Syntheses: Evolving Standards (RAMESES), evidence is selected not because it belongs to a preferred study design, but because it can contribute meaningfully to theory development or theory testing. Rigor is judged in relation to the credibility and trustworthiness of the data and the inferences drawn from them, while transparency in searching, selection, appraisal, analysis, and synthesis remains essential to review quality.

Recent methodological work in health professions education also underscores that realist analysis must move beyond descriptive lists of contexts, mechanisms, and outcomes. Rees and colleagues [63] argue that high-quality realist analysis requires retroductive theorizing, configurational analysis, and explicit attention to relevance, rigor, and richness. They also show that realist work is weakened when program activities are mistaken for mechanisms, when program theory remains underdeveloped, or when CMO configurations are presented without clearly explaining their causal relationships and contribution to theory refinement [63].

Accordingly, this protocol makes its realist logic explicit from the outset. It sets out a provisional initial program theory, an iterative and purposive search strategy, relevance and rigor-based inclusion decisions, and a retroductive analytic approach aimed at refining explanatory CMO configurations. The intention is not simply to catalog remediation strategies, but to explain how clinical skills remediation may succeed, falter, or produce only partial change in undergraduate and postgraduate medical training in LMIC settings. This realist review aims to explain how, why, for whom, and under what LMIC conditions clinical and procedural skills remediation produces improvement among undergraduate medical students and postgraduate medical trainees. Treating remediation as a complex social intervention whose outcomes are shaped by interactions between program resources, learner responses, supervisory relationships, and institutional contexts, the review will refine an initial program theory by identifying the mechanisms through which remediation works, the contexts that enable or suppress those mechanisms, and the CMO configurations that best account for variation in outcomes across settings.

## Methods

### Design

This study is a realist review protocol conducted in accordance with the RAMESES publication standards for realist syntheses and informed by the RAMESES quality standards for realist reviews [59-61]. A realist synthesis was chosen because the review seeks to explain how and why clinical and procedural skills remediation produces different outcomes across settings, rather than estimating an average intervention effect. In line with realist philosophy, remediation is treated as a complex social intervention whose outcomes arise when program resources interact with learner reasoning, supervisory relationships, and institutional and sociocultural contexts. The review is therefore designed to identify, test, and refine CMO configurations that explain why remediation succeeds, produces only partial or fragile improvement, or fails.

This protocol is submitted as a stage 1–registered report at the PRE-results stage (RR1-PRE) and is registered in PROSPERO (International Prospective Register of Systematic Reviews). At the time of submission, no formal database search, full screening, data extraction, or theory-testing synthesis has commenced. Protocol refinement, stakeholder-informed scoping work, preliminary literature familiarization, initial program theory development, and drafting of the search strategies have been completed. Any substantive amendments after formal review commencement will be dated, logged, updated in PROSPERO where appropriate, and reported transparently in the final review manuscript.

### Review Team, Expertise, and Responsibilities

The review team comprises MRU, MA, YL, PSU, RNH, FCU, CFC, AMJ, AIF, and YS. MRU and MA will serve as lead author and review coordinator, overseeing protocol integration, search documentation, evidence tables, version control, and preparation of the first manuscript draft. PSU and RNH will co-lead the realist methodological work, including refinement of the initial program theory, calibration of eligibility decisions, relevance-rigor appraisal, and higher-order CMO configuration interpretation. YS will provide senior oversight for realist-methodological decisions, unresolved adjudications, and final theory refinement. MA, YL, AMJ, and AIF will provide clinical-content adjudication, especially in relation to procedural-skill scope, clinical authenticity of interpretations, and transferability across training environments. AIF will also contribute to search strategy development and clinically meaningful refinement of the search terms. FCU and CFC will support full-text appraisal, data extraction, contextual coding, and educational interpretation of feedback, reflection, and learner-support mechanisms. All authors will contribute to the interpretation of findings, refinement of the final program theory, and revision of the manuscript. PSU and RNH bring previous experience with realist methodology in health professions education research; YS has published extensively in medical education assessment and supervision. All team members are based in Indonesian medical schools, which provides contextual strength for the anchor LMIC setting but may limit interpretive perspectives from other LMIC regions; this is partly mitigated by the planned multicountry stakeholder engagement and by drawing on the international evidence base during synthesis.

### Review Purpose and Focusing

This realist review aims to explain how, why, for whom, and under what LMIC conditions clinical and procedural skills remediation produces improvement, only fragile improvement, or no meaningful improvement in undergraduate medical students and postgraduate medical trainees. The review will refine an initial program theory by identifying the mechanisms through which remediation works, the contexts that enable or suppress those mechanisms, and the CMO configurations that best explain variation in outcomes across settings.

### Review Questions

This realist review addresses three primary questions:

1. How and why do clinical and procedural skills remediation work, or fail to work, for undergraduate medical students and postgraduate medical trainees?
2. For whom, in what LMIC contexts, and in what respects are remediation mechanisms activated, amplified, suppressed, or redirected?
3. What CMO configurations best explain variation in remediation outcomes across settings?

### Initial Program Theory

**Figure 1.**
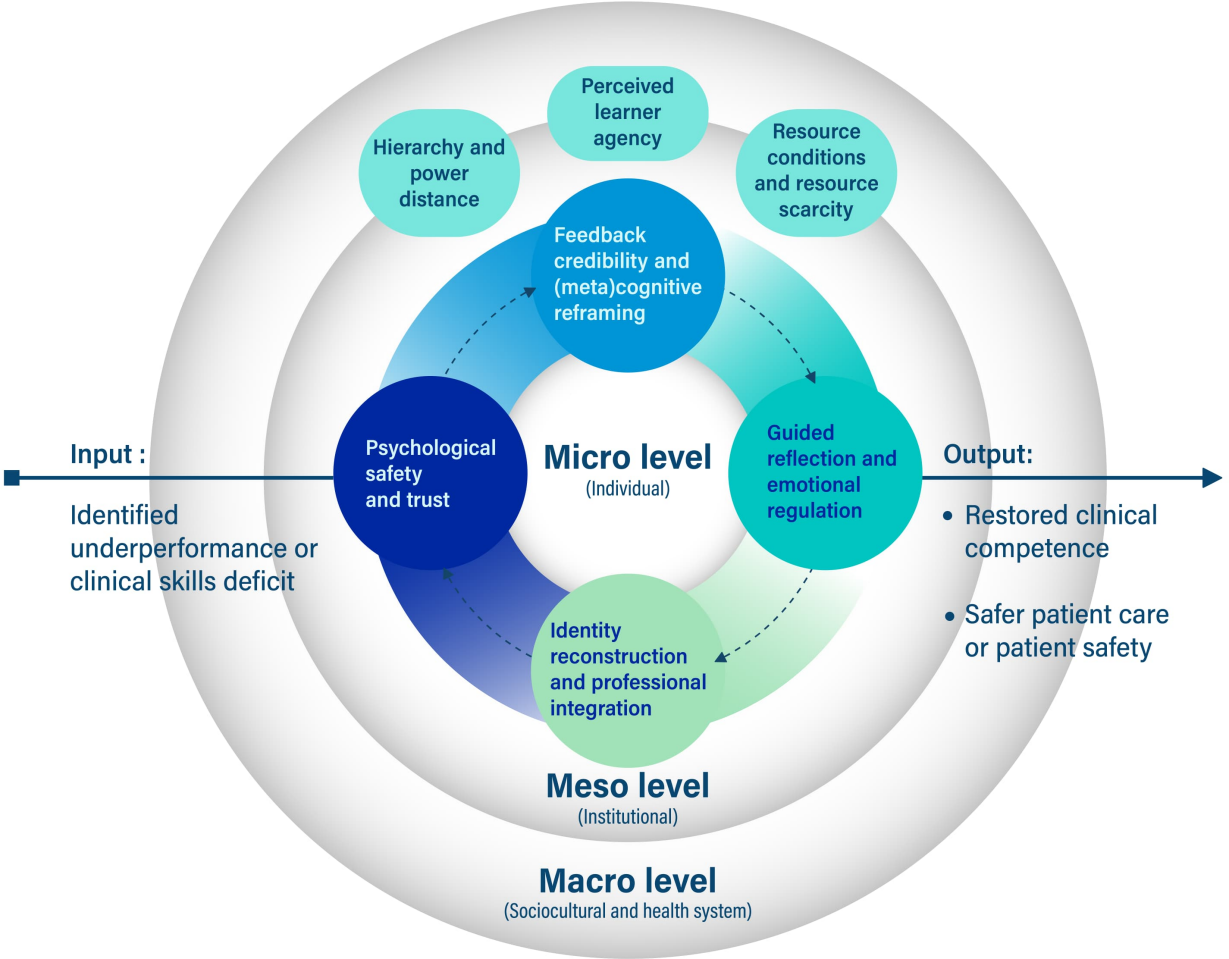
Initial program theory of clinical skills remediation in LMIC (low- and middle-income country) settings.

The initial program theory (IPT) proposes that clinical skills remediation is more likely to restore competence and improve patient-safety–related outcomes when 4 interacting mechanism domains are activated, such as psychological safety and trust, feedback credibility and metacognitive reframing, guided reflection and emotional regulation, and identity reconstruction and professional reintegration (Figure 1). These domains operate primarily at the microlevel of the learner but are shaped by mesolevel institutional factors and macrolevel sociocultural and health-system conditions. As shown in the Clinical Skills Remediation Framework (Multimedia Appendix 1), activation of these mechanisms is moderated by learner characteristics such as previous performance, self-efficacy, motivation, coping capacity, and previous experiences of feedback, as well as by institutional and contextual conditions such as faculty capacity, assessment infrastructure, cultural framing of remediation, hierarchy and power distance, resource constraints, health-system demands, regulatory expectations, and pandemic-related disruption.

The IPT was developed through 3 complementary and sequentially informing stages. In the first stage, a purposive scoping search of literature published between 2015 and 2024 was conducted across MEDLINE, ERIC, and PsycINFO using search terms related to medical underperformance, remediation, self-regulated learning, feedback literacy, psychological safety, and professional identity formation. From this search, five candidate explanatory domains were inductively identified on the basis of their recurrence across high-yield empirical and theoretical sources: (1) self-regulatory capacity, (2) feedback responsiveness, (3) psychological safety within the learning environment, (4) professional identity coherence, and (5) institutional and supervisory support structures. These candidate domains formed the provisional architecture of the IPT.

In the second stage, the candidate domains were stress-tested through advisory feedback sessions with 8 participants representing a purposive cross-section of clinical and educational roles, including clinicians actively involved in student remediation, medical educators with curriculum design responsibilities, and program coordinators with oversight of underperforming student pathways. To account for contextual variation in resource environments, institutional culture, and student population profiles, participants were drawn from 2 private universities and 4 public universities in urban settings, as well as 1 private university and 1 public university in rural settings. Sessions were conducted between October 2024 and January 2025, using 2 formats—individual semistructured interviews and focus group discussions—each lasting between 45 and 90 minutes. Structured around the candidate IPT domains, the sessions invited participants to affirm, contest, or refine proposed causal mechanisms; to identify contextual factors likely to activate or suppress those mechanisms; and to reflect on observed patterns of underperformance from their own practice. It should be noted that these sessions were advisory in nature and were not designed or conducted as formal qualitative research. No verbatim transcription or thematic analysis was performed; outputs were documented as field notes and used solely to interrogate and refine the provisional IPT, consistent with the role of practitioner consultation in realist synthesis methodology.

In the third stage, the refined domains were cross-checked against an integrative review of explanatory and empirical literature to ensure each domain carried both theoretical coherence and empirical grounding. Theoretical sources included Zimmermann’s model of self-regulated learning [64,65], Telio’s educational alliance model [66,67], and the framework by Cruess et al [68] for professional identity formation in health professionals, alongside empirical literature pertinent to remediation, feedback, psychological safety, and professional identity. The final IPT domains thus represent a convergence of inductive evidence from the literature, practitioner-informed contextual knowledge, and deductive theoretical reasoning. Consistent with realist methodology, the IPT is treated as provisional and subject to iterative refinement throughout the course of the review.

### Candidate Propositions

#### Overview

The following candidate propositions are derived from the initial program theory and will be tested, refined, or rejected during the synthesis. Each proposition is expressed in proto-CMO configuration form to make the review’s explanatory intent explicit.

#### Proposition 1: Psychological Safety

When remediation is framed as developmental rather than disciplinary (C), and supervisory relationships provide nonjudgmental support (C), learners are more likely to disclose genuine difficulties and engage with feedback (M: trust-based disclosure), leading to more accurate diagnosis of performance gaps and targeted intervention (O).

#### Proposition 2: Feedback Credibility

When feedback is specific, evidence-based, and delivered by a supervisor perceived as clinically credible (C), learners are more likely to accept the feedback as legitimate and recalibrate their self-assessment (M: metacognitive reframing), leading to focused remediation effort and measurable skills improvement (O).

#### Proposition 3: Guided Reflection and Emotional Regulation

When structured reflective activities are embedded within remediation and accompanied by emotional support (C), learners are more likely to process feedback without defensive withdrawal (M: regulated reflective processing), leading to long-term behavioral change rather than surface compliance (O).

#### Proposition 4: Identity Reconstruction

When the remediation process includes explicit attention to professional identity and reintegration into the clinical team (C), learners are more likely to restore a viable professional self-concept (M: identity repair and restored agency), leading to long-term competence maintenance and reduced attrition (O).

#### Proposition 5: LMIC Contextual Moderation—Hierarchy and Resource Scarcity

When LMIC conditions of strong hierarchical power distance, limited simulation access, and faculty scarcity are present (C), mechanisms of psychological safety and feedback credibility are weakened or redirected (M: suppressed disclosure; deferred to authority), producing fragile, compliance-driven improvement rather than deep behavioral change (O), even when program design includes components that work well in HIC settings.

#### Proposition 6: Stigma as Mechanism Suppressor

When remediation carries strong sociocultural stigma and is framed as failure rather than development (C), learners are more likely to conceal difficulties, avoid help-seeking, and disengage from the program (M: stigma-driven avoidance), leading to delayed identification, incomplete remediation, and continued underperformance (O).

These propositions are intentionally provisional. They function as testable starting points that will be refined, split, merged, or rejected as the synthesis proceeds.

### Eligibility Criteria

Eligibility will be applied at 2 levels—source-level eligibility for screening and inclusion, and fragment-level relevance-rigor judgment during analysis. At source level, documents will be included if they concern an explicit remediation response to identified underperformance, difficulty, or competence concern; involve undergraduate medical students, clinical clerks, interns, residents, registrars, or equivalent postgraduate medical trainees; address clinical skills, procedural skills, clinical competence, supervised performance, or closely related workplace-based performance domains; and contain empirical, documentary, or conceptually rich explanatory material capable of informing theory building, theory refinement, or theory testing. Full text must be available in English, Indonesian, or Bahasa Melayu, or be translatable adequately for analysis.

Documents will be excluded if they concern general teaching, assessment, coaching, or feedback with no remediation trigger linked to identified underperformance; focus exclusively on nonclinical academic performance; involve only nonmedical learners without clearly transferable theory-relevant insight for the medical education program theory; concern practicing doctors outside a training context and add no theory-relevant insight for trainee remediation; are duplicate reports with no unique contextual, mechanistic, or outcome data; are unavailable in full text after reasonable retrieval attempts; or contain only unsupported opinion, decontextualized assertion, or repeated summary without sufficient empirical or documentary grounding to support realist inference.

Eligible interventions include simulation-based remediation, supervised clinical practice, coaching or mentoring, structured feedback and reflection, peer-assisted remediation, and multicomponent programs. Studies from any relevant training context will be considered, including hospital, primary care, simulation, and community settings. No study design will be excluded in advance. Quantitative, qualitative, mixed methods, evaluative, policy, and theory-informed sources will be considered for inclusion where they offer data relevant to developing, refining, or testing the program theory. No absolute date restriction will be imposed at the search stage; however, interpretation will prioritize work from 2000 onward, while retaining earlier seminal texts where needed for theory development or citation chaining.

### Search Strategy

The search strategy has already been developed iteratively by the review team and is reported in Multimedia Appendix 2. Consistent with that appendix, the principal database searches will be structured around 3 concept blocks—population, remediation or performance-improvement phenomenon, and clinical or procedural skills context. The principal search will be run without a geographic filter to avoid prematurely excluding theory-relevant evidence. A supplementary LMIC overlay will then be applied separately to map LMIC-specific yield and support cross-context comparison.

Formal searches will be conducted in MEDLINE via PubMed, CINAHL, PsycINFO, ERIC, and Scopus. Before execution, the MEDLINE strategy will undergo Peer Review of Electronic Search Strategies–informed, independent information-specialist review. Preliminary scoping searches suggest an estimated retrieval of approximately 3000‐5000 titles for screening, which informed the decision to assign 3 independent screeners (MRU, YL, and AIF) at the title-and-abstract stage and to use Covidence’s machine learning–assisted prioritization to support efficient ordering of records for human review. MRU will lead database execution and citation management. PSU will comonitor search iteration and retrieval completeness. RNH and YS will oversee major search refinements if theory gaps emerge. MA and YL will advise on clinically meaningful synonym expansion for procedural and clinical-skills terminology. FCU and CFC will support supplementary searching of education-focused and gray literature sources.

To avoid false-negative retrieval of theory-relevant evidence, no geographic or LMIC filter was applied to the principal database search. LMIC relevance will instead be determined during title and abstract screening, full-text review, and data extraction based on study setting and country classification using the World Bank fiscal year 2026 framework [38]. A supplementary LMIC overlay search was explored for mapping purposes, but was not used as the primary retrieval strategy because it substantially reduced search sensitivity.

Supplementary searches will include ProQuest Dissertations and Theses Global, World Health Organization IRIS, the World Bank Open Knowledge Repository, World Federation for Medical Education documents, institutional repositories of LMIC medical schools, including Asosiasi Institusi Pendidikan Kedokteran Indonesia (Association of Indonesian Medical Schools) member institutions, and policy or guidance documents from relevant regulatory and professional bodies, including the Indonesian Medical Council (Konsil Kedokteran Indonesia) and comparable national medical councils in other LMIC jurisdictions. Forward and backward citation chaining will be undertaken for all included studies and influential index papers using Google Scholar and Scopus. Citations, lead authors, unpublished materials, scholar searches, theories, early examples, and related projects–style searching will be used, where appropriate, to locate companion papers, related projects, theory papers, program descriptions, and linked outputs around key study families. Stakeholder-recommended sources from recognized experts in medical education remediation will also be considered. These supplementary searches are planned not merely for comprehensiveness, but to locate data capable of seeding, refining, or testing specific parts of the IPT.

### Study Selection

Records will be managed in Covidence. After deduplication, title and abstract screening will be conducted independently by MRU, PSU, and FCU. Before full screening, MRU, PSU, and FCU will pilot the eligibility rules on a shared calibration set of 50 records and refine the decision rules until acceptable agreement is reached. Full-text review will then be conducted independently by MRU and CFC. Where ambiguity relates to clinical or procedural-skill scope, MA and YL will provide clinical adjudication. YS will adjudicate unresolved disagreements. Reasons for exclusion at the full-text stage will be logged systematically and reported in a PRISMA (Preferred Reporting Items for Systematic Reviews and Meta-Analyses) flow diagram. Covidence’s machine learning–assisted prioritization may be used to help order records for human review during title and abstract screening; however, all final inclusion and exclusion decisions will be made by human reviewers.

### Relevance and Rigor Appraisal

In keeping with realist methodology, selection and appraisal will not be treated as purely technical or sequential stages. Following full-text screening, documents and data fragments will be appraised iteratively for relevance and rigor. Relevance refers to whether a document, or a fragment within it, can contribute to theory building, refinement, or testing in relation to the review questions and IPT. Rigor refers to whether the method used to generate that particular piece of data is sufficiently credible and trustworthy for the inference being drawn from it.

PSU and RNH will lead the initial relevance-rigor appraisal, with MRU supporting documentation and YS adjudicating unresolved methodological disputes. Design-specific appraisal tools, such as Risk of Bias 2, Risk of Bias in Non-randomized Studies of Interventions, Critical Appraisal Skills Programme prompts, and Mixed Methods Appraisal Tool will be used only as aids to judgment rather than as automatic threshold devices. No single numeric score will determine inclusion or exclusion. Lower-rigor but high-relevance fragments may be retained cautiously, especially where they offer unique contextual or theoretical insight from underrepresented LMIC settings, and their limitations will be explicitly weighed during synthesis.

At the fragment level, unsupported author assertion, decontextualized opinion, or repeated summary without empirical or documentary grounding will not be used as confirmatory evidence. Conceptually rich commentaries and policy texts may nevertheless be retained for theory seeding or refinement if they illuminate how remediation is framed, justified, or operationalized in relevant settings.

### Data Extraction

A structured extraction form (Multimedia Appendix 3) will be used and refined iteratively. MRU and FCU will conduct a duplicate primary extraction. CFC will support linking of multiple reports into study families. PSU will audit a purposive subsample for consistency of configurational coding. MA and YL will review clinically complex or procedure-specific fragments to ensure that extracted inferences remain clinically defensible. YS will adjudicate unresolved extraction disputes and major recoding decisions.

Extraction will include document characteristics, learner group, setting, intervention features, contextual conditions, mechanism candidates, outcomes, candidate CMO configurations, and each source’s contribution to theory development or testing. Where multiple papers describe the same program, cohort, or intervention family, they will be linked and treated as a single analytic case, using the richest report as the anchor source and companion reports to supplement missing contextual or outcome detail.

Importantly, extraction will focus on explanation rather than summary alone. Each usable fragment will be coded according to the role it plays in the emerging explanation, such as context, program resource, participant reasoning, mechanism, outcome, implementation condition, or theory-related inference. This is intended to reduce the common realist error of presenting unlinked lists of contexts, mechanisms, and outcomes without explaining how they connect.

### Quality Control

Before full screening, appraisal, and extraction, the review team will undertake calibration exercises using a shared set of training papers. Agreement will be assessed pragmatically, and any systematic discrepancies will be discussed until common decision rules are established. PSU and RNH will lead quality control measures, which will include duplicate screening, duplicate relevance-rigor appraisal, duplicate extraction, dated search logs, study-family logs, theory-refinement memos, and a protocol-deviation log. The distinction between theory-seeding documents and theory-testing evidence will be maintained explicitly throughout the review.

### Data Synthesis

Synthesis will follow a realist logic of analysis using retroductive theorizing. The primary analytic unit will be the CMO configuration; a structured explanation of how particular contextual conditions shape whether and how program resources trigger mechanisms that generate specific outcomes. The review will move iteratively between theory and data, and between individual studies and the wider explanatory model, with the aim of building, testing, refuting, and refining the IPT rather than merely cataloging interventions or outcomes. The aim is not merely to catalog contexts, mechanisms, and outcomes but to explain the causal relationships between them, within and across CMO configurations [63].

Cross-case comparison will implement the following four analytical strategies throughout: (1) juxtaposition, comparing evidence from different studies and contexts to identify convergent and divergent patterns; (2) reconciliation, examining differences in results from similar situations to generate explanatory hypotheses; (3) adjudication, weighing methodological strengths and limitations to determine which evidence most reliably supports or challenges theoretical claims; and (4) consolidation, developing integrated explanations for why different outcomes occur in similar contexts. Analysis will proceed through four iterative stages:

1. Initial coding: Relevant text segments from included documents will be coded deductively against the initial program theory domains (psychological safety, feedback credibility, guided reflection, identity reconstruction) and against the cross-cutting sensitizing constructs of hierarchy and agency, while also allowing inductive identification of emergent themes. At this stage, each usable data fragment will be provisionally tagged for what role it plays in the explanation (context, mechanism, outcome, resource, or reasoning), consistent with RAMESES II guidance on realist analytic discipline.
2. CMO configuration development: Coded segments will be assembled into candidate CMO configurations, explicitly specifying the contextual conditions, the resources or opportunities offered by the remediation strategy, the participant reasoning or responses they appear to trigger, and the resulting outcomes.
3. Pattern recognition: CMO configurations will be compared across studies to identify demiregularities—recurrent patterns linking context, mechanism, and outcome across different settings.
4. Theory refinement: Demiregularities will be used to refine, extend, or challenge the initial program theory, producing a final program theory expressed as linked CMO configurations and a narrative explanation of how particular challenge-strategy pairings are expected to operate under different conditions.

The final program theory will be presented as a diagram and a narrative description of CMO configurations, distinguishing findings from LMIC and HIC settings where evidence permits. MRU, PSU, and RNH will lead first-pass coding and development of candidate CMO configurations. FCU and CFC will contribute to the consolidation of related configurations and educational interpretation of learner-support, feedback, and reflection processes. MA and YL will challenge clinical plausibility where proposed mechanisms, particularly those concerning supervised practice, patient safety, or procedural remediation, appear overextended or weakly supported. YS will chair theory-refinement meetings and confirm the final pattern-level explanation.

Analytic contingencies are prespecified as follows: if a source provides strong contextual description but limited mechanism data, it will be used primarily for context mapping; if it provides plausible participant reasoning but weak outcome linkage, it will be used to generate or refine candidate mechanisms rather than confirm CMO configurations; if multiple reports from the same program disagree, the richer and methodologically stronger report will anchor interpretation while discordant information is retained and examined; and if LMIC evidence is insufficient for a specific proposition, HIC evidence may be used as sensitizing material but any LMIC transfer claim will be presented as provisional. The exact correspondence between each review question, candidate proposition, sampling logic, and planned analysis is set out in the candidate propositions above.

NVivo (Lumivero) will be used to manage mechanism-rich qualitative coding and analytic memoing, while Microsoft Excel will be used for evidence tables, study-family tracking, and theory-development logs. Analytic memoing will document theory revisions, unresolved tensions, rationale for interpretive decisions, and links to substantive theory.

### Stakeholder Engagement

#### Overview

Following the literature-based synthesis and the generation of provisional refined CMO configurations, 2 sequential rounds of stakeholder engagement are planned. Each round serves a methodologically distinct function. The first is oriented toward interrogating and stress-testing the plausibility of the provisional program theory in light of practitioner experience; the second is oriented toward structured deliberation to consolidate, rank, and refine the CMO configurations before finalization. Collectively, these activities are designed to challenge and enrich the literature-derived program theory, not to replace the literature synthesis, and stakeholder input will not be treated as a standalone confirmatory dataset unless the protocol and ethics approvals are formally amended.

#### Round 1

The first round will be conducted as an online video workshop (90‐120 min, Zoom platform [Zoom Communications]) and will focus on presenting the provisional program theory and candidate CMO configurations derived from the synthesis. Participants will comprise approximately 12‐15 purposively sampled individuals representing a range of roles relevant to clinical skills remediation, including clinicians, medical educators, program directors, faculty supervisors, and trainees with direct experience of remediation pathways. Purposive sampling will prioritize diversity of institutional type, resource environment, and health-system context, with Indonesia serving as the primary anchor context given the review team’s situatedness, and additional LMIC representation sought across sub-Saharan Africa, Southeast Asia, South Asia, and North Africa.

Recruitment will be coordinated through established medical and health professions education networks selected on the basis of the following four criteria: (1) formal legal registration and institutional legitimacy; (2) established membership bases with demonstrable reach into clinical and health professions education communities; (3) previous engagement in international collaborative research or policy activities; and (4) logistical feasibility for online multicountry participation. Networks engaged will include the Indonesian Association for Medical and Health Professions Education and the Indonesian Medical Schools Association; the Southern African Association of Health Educationalists for sub-Saharan African representation; the Southeast Asian Regional Association for Medical Education for broader Southeast Asian representation; and Foundation for Advancement of International Medical Education and Research Regional Institute alumni to extend reach into South Asian, Latin American, and North African LMIC contexts. Recruitment will be supplemented by direct invitations from review team members and snowball referrals when network pathways are insufficient to reach target participant profiles, particularly for trainees and learners with lived remediation experience.

#### Round 2

The second round will use a modified nominal group technique (NGT), adapted for hybrid asynchronous and synchronous online delivery to accommodate cross-timezone participation across LMIC settings. Approximately 15‐20 participants will be engaged, including a subset drawn from round 1 for continuity and supplemented by additional participants with policy-level or program governance responsibilities. This round will be specifically oriented toward testing the CMO configurations refined following round 1, with participants asked to rank, contest, and elaborate proposed configurations in light of their institutional and contextual experience. The NGT format was selected in preference to Delphi methods—which risk conflating consensus with validity over iterative anonymous rounds—and to open focus group formats, which can suppress minority perspectives in contexts characterized by hierarchical power relations and status asymmetries, a pattern documented in LMIC academic and clinical cultures. The NGT’s structured sequencing of individual response, round-robin sharing, clarification, and ranked voting produces an explicit record of areas of agreement, disagreement, and residual uncertainty that can be directly incorporated into the synthesis narrative.

Across both rounds, multicountry LMIC representation is treated not merely as an inclusivity measure but as a substantive methodological requirement. The review’s central analytical interest—how context moderates the activation of mechanisms and the generation of outcomes—cannot be adequately addressed through a single-country or single-system stakeholder perspective. Heterogeneity of context is therefore a design feature rather than a logistical accommodation. If stakeholder input is subsequently judged to constitute a formal qualitative dataset warranting independent analysis beyond its advisory function, this will be documented as a protocol amendment and governed by the appropriate additional ethics approval processes.

### Ethical Considerations

This realist review involves the synthesis of published and publicly accessible materials and therefore does not require ethical approval for the literature review component. Any stakeholder engagement involving human participants will be undertaken only after obtaining formal approval from the relevant Research Ethics Committee, and all participants will provide informed consent. The literature-synthesis component remains nonhuman subjects research, whereas the stakeholder-engagement component will be governed by the approved ethics framework. Findings will be disseminated through peer-reviewed publication, conference presentation, and supplementary materials to ensure transparency of review conduct and reporting.

## Results

Funding for this review has been secured through Badan Riset dan Inovasi Nasional (National Research and Innovation Agency of Indonesia), and the protocol has been registered in PROSPERO (CRD42023447029). As of April 1, 2026, no formal database searching, full screening, data extraction, or theory-testing synthesis has commenced. Protocol refinement, stakeholder-informed scoping work, preliminary literature familiarization, and initial program theory development have been completed. Formal database and gray literature searching will begin in 2026, followed by duplicate screening, relevance and rigor appraisal, data extraction, and iterative realist synthesis through 2026‐2027. The review is expected to generate a refined program theory, a set of explanatory CMO configurations, and an evidence-informed account of how and why clinical and procedural skills remediation succeeds, falters, or produces only partial change in LMIC training contexts. These findings are intended to inform future remediation design, implementation, and policy in undergraduate and postgraduate medical training. The primary review manuscript is targeted for submission in 2027. Any substantive amendments after formal review commencement will be logged in PROSPERO and reported transparently in the final review manuscript.

## Discussion

### Anticipated Findings

This realist review is expected to produce a refined, transferable program theory explaining how and why clinical and procedural skills remediation works, for whom, and under what conditions in undergraduate and postgraduate medical training, with particular attention to LMIC settings. Rather than generating a single estimate of effectiveness, the review is anticipated to identify a set of explanatory CMO configurations showing how remediation succeeds, produces only partial or fragile improvement, or fails under different contextual conditions.

Based on the initial program theory and formative scoping work, the review is expected to examine whether remediation is more likely to generate meaningful improvement when remediation is framed as developmental rather than disciplinary, when supervisory relationships provide psychological safety and credible, specific feedback, when structured reflection is embedded within the remediation process, when learners retain some sense of agency in planning and enacting change, and when assessment systems support longitudinal feedback and supervised improvement rather than relying on high-stakes judgment alone. The review is also likely to clarify how LMIC-specific conditions, including resource scarcity, limited access to simulation and supervised practice, faculty-capacity constraints, sociocultural stigma, and hierarchical clinical environments, may amplify, suppress, or redirect these mechanisms in ways not adequately captured in existing HIC-dominated reviews.

### Comparison With Previous Work

This protocol builds directly on the RESTORE realist review [58], which conceptualized remediation as a behavior change process and identified 29 CMO configurations across 141 studies of practicing doctors. Our review extends RESTORE in 3 directions; it focuses on undergraduate and postgraduate trainees rather than qualified practitioners, it foregrounds LMIC contexts where resource constraints, hierarchy, and stigma may substantially alter mechanism activation, and it draws on recent methodological advances in realist analysis, particularly the quality criteria proposed by Rees and colleagues [63] and the mechanism-resource versus mechanism-reasoning distinction articulated by Dalkin et al [69]. Our protocol also complements the Best Evidence Medical Education systematic reviews [53,54] and scoping review by Cheong et al [57] by shifting the analytic focus from cataloging intervention components to explaining the causal configurations through which remediation works or fails.

### Strengths and Limitations

This protocol has several strengths. First, it adopts a theory-driven and mechanism-focused realist approach suited to explaining how and why remediation outcomes vary across settings rather than merely describing intervention components. Second, it explicitly prioritizes LMIC contexts, thereby addressing an important gap in the literature base that remains dominated by high-income settings. Third, it includes diverse forms of evidence, allowing the review to draw on qualitative, quantitative, mixed methods, documentary, and gray literature sources where these contribute to theory building, refinement, or testing. Fourth, it is strengthened by stakeholder-informed scoping, an explicit initial program theory, and a prespecified analytic logic that seeks to avoid common realist errors, such as conflating program activities with mechanisms or presenting unlinked lists of contexts, mechanisms, and outcomes [63].

Additional strengths include the protocol’s explicit dialogue with previous realist work on remediation, its treatment of hierarchy, stigma, learner agency, and resource scarcity as cross-cutting contextual influences rather than peripheral background variables, and its stage 1–registered report logic, which prespecifies candidate propositions, exclusion rules, quality checks, and analytic contingencies before formal review commencement.

Several limitations should also be acknowledged. The evidence base is likely to be heterogeneous in intervention design, remediation triggers, learner groups, settings, and outcome reporting, which may limit direct comparability across sources. Some mechanisms central to remediation may be only partially visible in published reports and may therefore require cautious retroductive inference rather than direct empirical statement. Publication bias, availability bias, and indexing bias may also reduce access to relevant data, particularly where local LMIC practices are poorly documented, unpublished, or not indexed in major databases. In addition, where LMIC-specific evidence is sparse for particular propositions, the review may need to use HIC evidence as sensitizing material, meaning that some transfer claims will remain provisional rather than definitive.

### Future Directions

If the refined program theory proves sufficiently robust, it may inform the design and evaluation of remediation interventions tailored to LMIC contexts. Future empirical work could test specific CMO configurations through realist evaluation of remediation programs in Indonesian and other LMIC medical schools. The candidate propositions developed in this protocol, particularly those concerning hierarchy-mediated suppression (proposition 5) and stigma-driven avoidance (proposition 6), may also serve as a foundation for context-sensitive implementation research that examines how remediation systems can be adapted to function within, rather than against, local sociocultural structures.

### Conclusion

Clinical skills remediation is a complex social intervention whose outcomes depend not only on program design but on how learners, supervisors, and institutions interact within specific contextual conditions. Existing reviews have cataloged remediation strategies and documented their broad effectiveness, yet the field still lacks a coherent explanatory account of how and why remediation works for trainees in resource-constrained LMIC settings. This realist review protocol addresses that gap by specifying a theory-driven, mechanism-focused approach grounded in 6 candidate propositions that will be tested, refined, or rejected through retroductive synthesis of international evidence.

By moving beyond description toward explanation, this review aims to produce a refined program theory that clarifies the conditions under which psychological safety, feedback credibility, guided reflection, and identity reconstruction are activated or suppressed, and how LMIC-specific factors, such as hierarchy, stigma, faculty scarcity, and resource constraints, moderate these mechanisms. The resulting evidence-informed framework is intended to guide the design of remediation systems that are contextually grounded, educationally sound, and capable of restoring safe clinical performance across both undergraduate and postgraduate medical training.

## Supplementary material

10.2196/89550 Multimedia Appendix 1 Initial program theory diagram (Clinical Skills Remediation Framework).

10.2196/89550 Multimedia Appendix 2 Database search strategies.

10.2196/89550 Multimedia Appendix 3 Data extraction form and coding fields.

## Abbreviations

CMO: context-mechanism-outcome
HIC: high-income country
IPT: initial program theory
LMIC: low- and middle-income country
NGT: nominal group technique
PRISMA: Preferred Reporting Items for Systematic Reviews and Meta-Analyses
PROSPERO: International Prospective Register of Systematic Reviews
RAMESES: Realist And Meta-narrative Evidence Syntheses: Evolving Standards
RESTORE: Realist Synthesis of Doctor Remediation
