# Supplementary material for: Challenges, Strategies, and Explanatory Mechanisms in Clinical Skills Remediation Programs in Undergraduate and Postgraduate Medical Education in Low- and Middle-Income Countries: Realist Review Protocol
Source: JMIR Res Protoc. 2026 Jun 2;15:e89550. doi: 10.2196/89550 (PMC13229394; doi:10.2196/89550)
Supplement: Multimedia Appendix 1 [file resprot-v15-e89550-s001.docx]

*Challenges, Strategies, and Explanatory Mechanisms in Clinical Skills Remediation Programs in Undergraduate and Postgraduate Medical Education in Low- and Middle-Income Countries: A Realist Review Protocol*

PROSPERO Registration: CRD42023447029

# Overview

This appendix provides an interpretive guide to the Clinical Skills Remediation Framework shown in Figure 1. The framework is intended as a heuristic representation of the review team’s starting assumptions about how remediation may operate in clinical and procedural skills training in low- and middle-income country settings. It is not presented as a fixed causal model or a claim of linear effectiveness. Rather, it functions as a provisional map of how different forms of underperformance may be addressed through interacting learner-level, institutional, and wider contextual processes, and how those processes may contribute to restored competence and safer patient care under some conditions but not others.


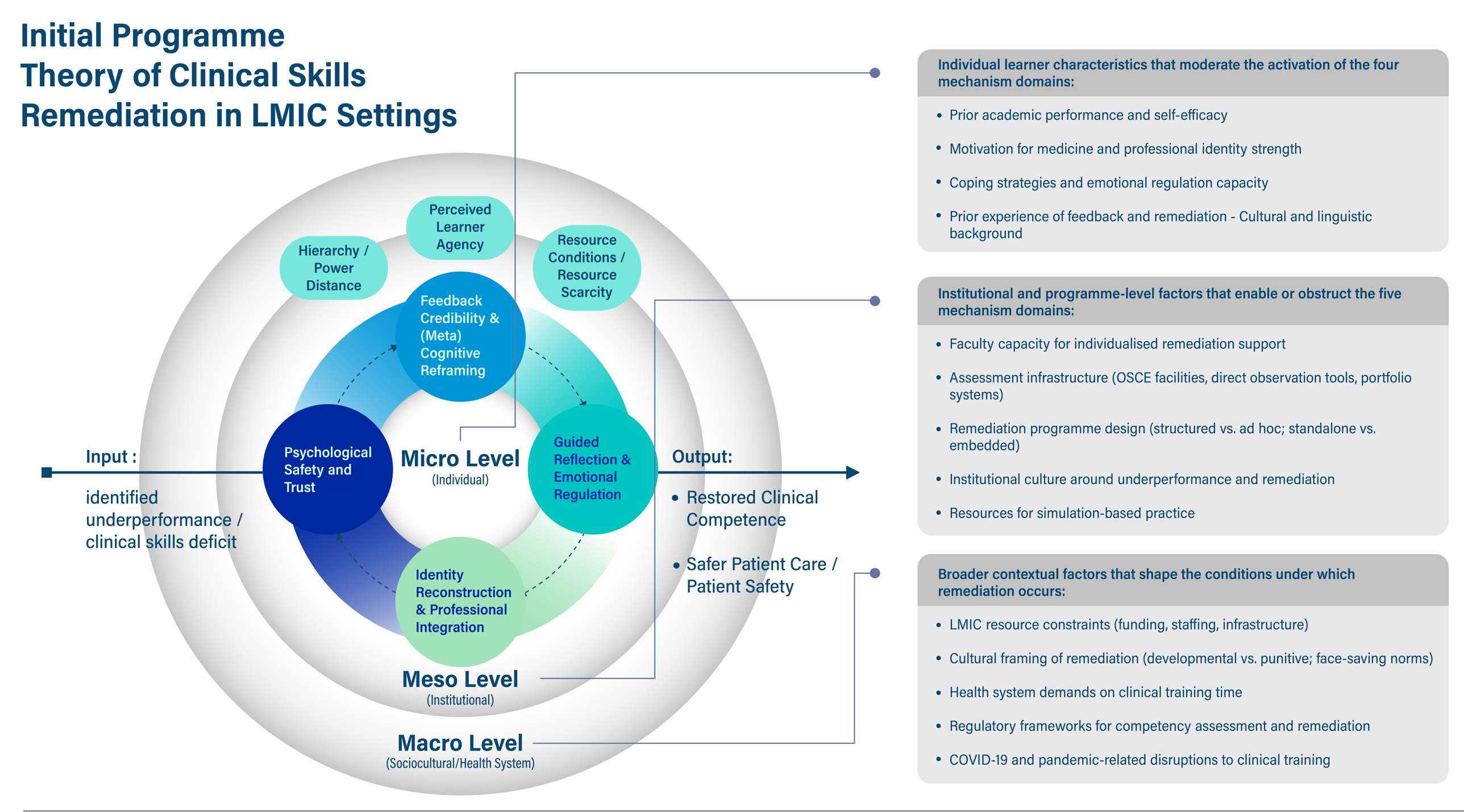


**Figure 1. Initial Programme Theory of Clinical Skills Remediation in LMIC Settings.**

The figure is read broadly from left to right. On the left, the “input” represents identified underperformance or a clinical skills deficit that triggers a remediation response. On the right, the “output” represents the intended educational and clinical consequences of successful remediation, namely restored clinical competence and safer patient care. The framework does not assume that movement from input to output is automatic. Instead, it depicts remediation as a contingent process in which outcomes depend on whether key explanatory processes are activated, supported, or constrained within a given setting.

At the centre of the figure is the **micro level**, which represents the learner-facing space in which remediation is most directly experienced. The four central domains are positioned as interacting explanatory processes rather than sequential steps. Their circular arrangement is deliberate: it indicates that remediation is expected to be iterative, recursive, and mutually reinforcing rather than strictly linear. In practice, progress in one domain may support movement in another, while disruption in one domain may weaken the others.

The first domain, **psychological safety and trust**, occupies a foundational position because disclosure of difficulty, engagement with feedback, and willingness to attempt corrective action are unlikely to occur in the absence of a sufficiently safe relational environment. The second domain, **feedback credibility and metacognitive reframing**, refers to the learner’s recognition that the performance gap is real, intelligible, and potentially remediable. The third domain, **guided reflection and emotional regulation**, reflects the expectation that feedback alone is insufficient unless learners are able to process it, regulate its emotional impact, and convert it into deliberate action. The fourth domain, **identity reconstruction and professional integration**, represents the possibility that remediation is not simply technical retraining but may also involve restoring a disrupted sense of competence, legitimacy, and belonging within the profession. The dotted arrows between these domains indicate reciprocal influence rather than fixed temporal order.

Several influences are positioned near the boundary of the central field rather than within it. These include **hierarchy and power distance**, **perceived learner agency**, and **resource conditions or scarcity**. Their location is important. They are not treated here as stand-alone intervention components, nor are they framed as isolated background descriptors. Instead, they are represented as cross-cutting conditions that may shape whether the central processes are activated, muted, delayed, or redirected. For example, a learner may receive technically adequate feedback, but the explanatory force of that feedback may be weakened if hierarchy discourages open discussion, if agency is low, or if remediation opportunities are materially constrained.

Surrounding the micro level are the **meso** and **macro** layers. These concentric fields indicate that remediation unfolds within nested systems rather than within the learner alone. The **meso level** represents the institutional and programme environment in which remediation is organised, enacted, and interpreted. This includes the local assessment system, the way remediation is designed, the availability of supervisors and structured support, and the prevailing institutional culture around underperformance. The **macro level** refers to broader sociocultural and health-system conditions, including workforce pressures, regulatory expectations, prevailing norms about error and competence, and resource limitations that may shape both what remediation is possible and how it is socially understood. The layered structure is intended to show permeability rather than separation: influences from these outer levels are expected to enter the learner-facing process continuously.

The three shaded boxes on the right side of the figure unpack these broader influences into analytically useful groupings. The upper box identifies **individual learner characteristics** that may moderate how remediation is received and acted upon, such as prior performance, self-efficacy, motivation, coping capacity, and previous experiences of feedback. The middle box identifies **institutional and programme-level factors**, such as faculty capacity, assessment infrastructure, remediation design, institutional culture, and access to simulation-based practice. The lower box identifies **broader contextual conditions**, including resource constraints, cultural framing of remediation, health-system demands, regulatory structures, and disruption related to the COVID-19 period. These categories are shown separately for readability, but they should not be interpreted as independent silos; in practice, they are likely to interact.

This visual framework should therefore be interpreted as a **starting architecture for synthesis**, not as a completed theory. It is designed to support later stages of the realist review by guiding searching, data extraction, and theory refinement. It also clarifies an important analytic boundary: programme activities such as simulation, supervised practice, coaching, structured feedback, or reflective tasks are not assumed to be mechanisms in themselves. In this framework, they are treated as programme resources or opportunities — consistent with the distinction between mechanism-resource and mechanism-reasoning (Dalkin et al., 2015) — whose explanatory significance depends on how they are encountered, interpreted, and acted upon in context. Six candidate propositions derived from this framework are set out in the main protocol manuscript to make the review’s explanatory intent testable from the outset. Likewise, the outputs shown on the right side of the figure should be understood as intended outcomes rather than guaranteed consequences.

Finally, the figure is deliberately simplified. It does not attempt to represent every possible pathway, nor does it imply that all remediation cases will involve all domains to the same degree. Some cases may be driven primarily by issues of feedback legitimacy and learner agency; others may be shaped more strongly by stigma, weak infrastructure, or the absence of safe supervisory relationships. The value of the framework lies in making these assumptions explicit so that they can be examined, challenged, refined, or rejected during the review process. In that sense, the appendix is not an extension of the results; it is a clarification of how the initial programme theory is organised visually and how it should be read analytically.
