# Supplementary material for: Challenges, Strategies, and Explanatory Mechanisms in Clinical Skills Remediation Programs in Undergraduate and Postgraduate Medical Education in Low- and Middle-Income Countries: Realist Review Protocol
Source: JMIR Res Protoc. 2026 Jun 2;15:e89550. doi: 10.2196/89550 (PMC13229394; doi:10.2196/89550)
Supplement: Multimedia Appendix 2 [file resprot-v15-e89550-s002.docx]

*Challenges, Strategies, and Explanatory Mechanisms in Clinical Skills Remediation Programs in Undergraduate and Postgraduate Medical Education in Low- and Middle-Income Countries: A Realist Review Protocol*

**PROSPERO Registration: CRD42023447029**

# Overview

**The search architecture is structured around three core concepts:** (1) Population: undergraduate and postgraduate medical learners; (2) Intervention/Phenomenon: remediation and related performance-improvement processes; and (3) Context: clinical or procedural skills, clinical competence, simulation, and supervised practice.

The principal search will be conducted without an LMIC geographic filter to maximise sensitivity and avoid premature exclusion of theory-relevant evidence. Study setting and LMIC classification (per World Bank fiscal year 2026 income classifications) will be determined during title/abstract screening, full-text review, and data extraction. A supplementary LMIC overlay will be applied separately to map LMIC-specific yield; it will not determine eligibility for inclusion.

The search strategies below were developed iteratively by the review team (MRU, YL, PSU, and AIF) using controlled vocabulary and free-text terms tailored to each database platform, in consultation with an information specialist. Before execution, the MEDLINE strategy will undergo independent review using the PRESS (Peer Review of Electronic Search Strategies) checklist. Preliminary scoping searches suggest an estimated retrieval of approximately 3,000–5,000 titles for screening across all databases. Strategy development took place between January and April 2026. The draft strategy was revised after pilot testing and internal review to improve specificity and reduce avoidable noise.

Revisions included:

- narrowing broad trainee terms such as ‘resident*’ and ‘registrar*’ to ‘medical resident*’ and ‘medical registrar*’
- removing overly broad terms such as ‘academic support’
- adding spelling and wording variants such as ‘remedial programme*’, ‘underperform*’, and ‘academic difficult*’
- keeping the LMIC filter separate from the principal search

# 1. MEDLINE (via PubMed)

| **Database details** | Database: MEDLINE via PubMed  Host: National Library of Medicine  Coverage: 2000 to present  Limits planned at execution: English, Indonesian, and Bahasa Melayu |
| --- | --- |
| **Main search strategy** | See code block below. |

**Main search string**

1. ("medical student*"[tiab] OR "undergraduate medical"[tiab] OR "medical trainee*"[tiab] OR "medical resident*"[tiab] OR "medical registrar*"[tiab] OR "postgraduate medical"[tiab] OR "medical education"[tiab] OR "medical intern*"[tiab] OR "clinical clerk*"[tiab] OR "Education, Medical, Undergraduate"[Mesh] OR "Education, Medical, Graduate"[Mesh] OR "Internship and Residency"[Mesh])
2. (remediation[tiab] OR "remedial program*"[tiab] OR "remedial programme*"[tiab] OR "remedial training"[tiab] OR "performance improvement"[tiab] OR underperform*[tiab] OR "academic difficult*"[tiab] OR "academic failure"[tiab] OR "skills remediation"[tiab] OR "clinical remediation"[tiab] OR "fitness to practise"[tiab] OR "fitness to practice"[tiab] OR "remedial intervention*"[tiab] OR "remedial education"[tiab] OR "performance deficien*"[tiab] OR "competence restoration"[tiab])
3. ("clinical skill*"[tiab] OR "procedural skill*"[tiab] OR "clinical performance"[tiab] OR OSCE[tiab] OR "objective structured clinical examination"[tiab] OR simulation[tiab] OR "simulation-based"[tiab] OR "clinical simulation"[tiab] OR "clinical training"[tiab] OR "supervised practice"[tiab] OR "Clinical Competence"[Mesh] OR "Simulation Training"[Mesh])
4. 1 AND 2 AND 3

Supplementary LMIC overlay (to be applied separately for mapping LMIC-specific yield, not for the principal search execution).

AND
("low-income countr*"[tiab]

OR "middle-income countr*"[tiab]

OR "low- and middle-income"[tiab]

OR LMIC [tiab]

OR "developing countr*"[tiab]

OR "resource-limited"[tiab]

OR "resource-constrained"[tiab]

OR "sub-Saharan Africa"[tiab]

OR "North Africa"[tiab]

OR "Southeast Asia"[tiab]

OR "South Asia"[tiab]

OR "Latin America"[tiab])

# 2. CINAHL (via EBSCOhost)

**Main search string**

((MH "Students, Medical+")

OR (MH "Education, Medical, Undergraduate+")

OR (MH "Education, Medical, Graduate+")

OR TI ("medical student*" OR "undergraduate medical" OR "medical trainee*" OR "medical resident*" OR "medical registrar*" OR "postgraduate medical" OR "medical education" OR "medical intern*" OR "clinical clerk*")

OR AB ("medical student*" OR "undergraduate medical" OR "medical trainee*" OR "medical resident*" OR "medical registrar*" OR "postgraduate medical" OR "medical education" OR "medical intern*" OR "clinical clerk*"))

AND
(TI (remediation OR "remedial program*" OR "remedial programme*" OR "remedial training" OR "performance improvement" OR underperform* OR "academic difficult*" OR "academic failure" OR "skills remediation" OR "clinical remediation" OR "fitness to practise" OR "fitness to practice" OR "remedial intervention*" OR "remedial education"

OR "performance deficien*" OR "competence restoration")

OR AB (remediation OR "remedial program*" OR "remedial programme*" OR "remedial training" OR "performance improvement" OR underperform* OR "academic difficult*" OR "academic failure" OR "skills remediation" OR "clinical remediation" OR "fitness to practise" OR "fitness to practice" OR "remedial intervention*" OR "remedial education"

OR "performance deficien*" OR "competence restoration"))

AND
((MH "Clinical Competence+")

OR (MH "Simulation Training+")

OR TI ("clinical skill*" OR "procedural skill*" OR "clinical competence" OR "clinical performance" OR OSCE OR "objective structured clinical examination" OR simulation OR "simulation-based" OR "clinical simulation" OR "clinical training" OR "supervised practice")

OR AB ("clinical skill*" OR "procedural skill*" OR "clinical competence" OR "clinical performance" OR OSCE OR "objective structured clinical examination" OR simulation

OR "simulation-based" OR "clinical simulation" OR "clinical training" OR "supervised practice"))

Supplementary LMIC overlay (to be applied separately for mapping LMIC-specific yield, not for the principal search execution).

AND
(TI ("low-income countr*" OR "middle-income countr*" OR "low- and middle-income" OR LMIC OR "developing countr*" OR "resource-limited" OR "resource-constrained" OR "sub-Saharan Africa" OR "North Africa" OR "Southeast Asia" OR "South Asia" OR "Latin America")

OR AB ("low-income countr*" OR "middle-income countr*" OR "low- and middle-income"

OR LMIC OR "developing countr*" OR "resource-limited" OR "resource-constrained" OR "sub-Saharan Africa" OR "North Africa" OR "Southeast Asia" OR "South Asia" OR "Latin America"))

# 3. PsycINFO (via Ovid)

**Main search string**

(exp Medical Students/

OR exp Medical Education/

OR ("medical student*" or "undergraduate medical" or "medical trainee*" or "medical resident*" or "medical registrar*" or "postgraduate medical" or "medical education" or "medical intern*" or "clinical clerk*").ti,ab.)

AND
((remediation or "remedial program*" or "remedial programme*" or "remedial training" or "performance improvement" or underperform* or "academic difficult*" or "academic failure" or "skills remediation" or "clinical remediation" or "fitness to practise" or "fitness to practice" or "remedial intervention*" or "remedial education" or "performance deficien*" or "competence restoration").ti,ab.)

AND
(("clinical skill*" or "procedural skill*" or "clinical competence" or "clinical performance" or OSCE or "objective structured clinical examination" or simulation or "simulation-based" or "clinical simulation" or "clinical training" or "supervised practice").ti,ab.)

Supplementary LMIC overlay (to be applied separately for mapping LMIC-specific yield, not for the principal search execution).

AND
(("low-income countr*" or "middle-income countr*" or "low- and middle-income" or LMIC or "developing countr*" or "resource-limited" or "resource-constrained" or "sub-Saharan Africa" or "North Africa" or "Southeast Asia" or "South Asia" or "Latin America").ti,ab.)

# 4. ERIC (via EBSCOhost or ProQuest)

**Main search string**

(DE "Medical Students"

OR DE "Medical Education"

OR TI ("medical student*" OR "undergraduate medical" OR "medical trainee*" OR "medical resident*" OR "medical registrar*" OR "postgraduate medical" OR "medical education" OR "medical intern*" OR "clinical clerk*")

OR AB ("medical student*" OR "undergraduate medical" OR "medical trainee*" OR "medical resident*" OR "medical registrar*" OR "postgraduate medical" OR "medical education" OR "medical intern*" OR "clinical clerk*"))

AND
(TI (remediation OR "remedial program*" OR "remedial programme*" OR "remedial training" OR "performance improvement" OR underperform* OR "academic difficult*" OR "academic failure" OR "skills remediation" OR "clinical remediation" OR "fitness to practise" OR "fitness to practice" OR "remedial intervention*" OR "remedial education"

OR "performance deficien*" OR "competence restoration")

OR AB (remediation OR "remedial program*" OR "remedial programme*" OR "remedial training" OR "performance improvement" OR underperform* OR "academic difficult*" OR "academic failure" OR "skills remediation" OR "clinical remediation" OR "fitness to practise" OR "fitness to practice" OR "remedial intervention*" OR "remedial education"

OR "performance deficien*" OR "competence restoration"))

AND
(DE "Clinical Experience"

OR TI ("clinical skill*" OR "procedural skill*" OR "clinical competence" OR "clinical performance" OR OSCE OR "objective structured clinical examination" OR simulation

OR "simulation-based" OR "clinical simulation" OR "clinical training" OR "supervised practice")

OR AB ("clinical skill*" OR "procedural skill*" OR "clinical competence" OR "clinical performance" OR OSCE OR "objective structured clinical examination" OR simulation

OR "simulation-based" OR "clinical simulation" OR "clinical training" OR "supervised practice"))

Supplementary LMIC overlay (to be applied separately for mapping LMIC-specific yield, not for the principal search execution).

AND
(TI ("low-income countr*" OR "middle-income countr*" OR "low- and middle-income" OR LMIC OR "developing countr*" OR "resource-limited" OR "resource-constrained" OR "sub-Saharan Africa" OR "North Africa" OR "Southeast Asia" OR "South Asia" OR "Latin America")

OR AB ("low-income countr*" OR "middle-income countr*" OR "low- and middle-income"

OR LMIC OR "developing countr*" OR "resource-limited" OR "resource-constrained" OR "sub-Saharan Africa" OR "North Africa" OR "Southeast Asia" OR "South Asia" OR "Latin America"))

# 5. Scopus (via Elsevier)

**Main search string**

(TITLE-ABS-KEY("medical student*" OR "undergraduate medical" OR "medical trainee*" OR "medical resident*" OR "medical registrar*" OR "postgraduate medical" OR "medical education" OR "medical intern*" OR "clinical clerk*"))
AND
(TITLE-ABS-KEY(remediation OR "remedial program*" OR "remedial programme*" OR "remedial training" OR "performance improvement" OR underperform* OR "academic difficult*" OR "academic failure" OR "skills remediation" OR "clinical remediation" OR "fitness to practise" OR "fitness to practice" OR "remedial intervention*" OR "remedial education" OR "performance deficien*" OR "competence restoration"))
AND
(TITLE-ABS-KEY("clinical skill*" OR "procedural skill*" OR "clinical competence" OR "clinical performance" OR OSCE OR "objective structured clinical examination" OR simulation OR "simulation-based" OR "clinical simulation" OR "clinical training" OR "supervised practice"))

Supplementary LMIC overlay (to be applied separately for mapping LMIC-specific yield, not for the principal search execution).

AND
(TITLE-ABS-KEY("low-income countr*" OR "middle-income countr*" OR "low- and middle-income" OR LMIC OR "developing countr*" OR "resource-limited" OR "resource-constrained" OR "sub-Saharan Africa" OR "North Africa" OR "Southeast Asia" OR "South Asia" OR "Latin America"))

# 6. Grey Literature and Supplementary Sources

In addition to the five principal database searches above, the following grey literature and supplementary sources will be searched using targeted keyword combinations derived from the three concept blocks (population, remediation/phenomenon, clinical skills context). These searches are planned not merely for comprehensiveness, but to locate data capable of seeding, refining, or testing specific parts of the initial programme theory.

**Dissertations and theses:** ProQuest Dissertations and Theses Global

**International health organisations:** WHO IRIS (Institutional Repository for Information Sharing); World Bank Open Knowledge Repository

**Medical education bodies:** World Federation for Medical Education (WFME) documents and standards; Konsil Kedokteran Indonesia (KKI, Indonesian Medical Council) guidelines and policy documents; comparable national medical council publications from other LMIC jurisdictions

**Institutional repositories:** Institutional repositories of LMIC medical schools, including AIPKI (Asosiasi Institusi Pendidikan Kedokteran Indonesia) member institutions

**Expert recommendations:** Stakeholder-recommended sources from recognised experts in medical education remediation, identified through the review team’s professional networks (I AM-HPE, AIPKI) and purposive invitation

# 7. Supplementary Search Methods

The following supplementary search methods will be used to identify additional theory-relevant evidence not captured by the principal database searches:

**Forward and backward citation chaining:** Will be undertaken for all included studies and influential index papers using Google Scholar and Scopus.

**CLUSTER-style searching:** Will be used, where appropriate, to locate companion papers, related projects, theory papers, programme descriptions, and linked outputs around key study families.

**Purposive theory-driven searching:** If specific aspects of the initial programme theory (e.g., psychological safety in hierarchical clinical environments, stigma and remediation framing in collectivist cultures) remain insufficiently addressed by the principal search, targeted supplementary searches will be conducted using Google Scholar and relevant disciplinary databases to locate theoretical, empirical, or policy literature that can inform those specific propositions.

# Notes

Consistent with RAMESES standards for realist synthesis, the search strategy is designed to be iterative. The strategies presented above represent the planned principal database searches. Additional searches may be conducted as the review progresses if emerging programme theory reveals gaps in the evidence base that require targeted retrieval. All search iterations will be dated, logged, and reported transparently in the final review manuscript. The MEDLINE strategy will undergo PRESS-informed independent review by a qualified information specialist before formal execution. Language limits (English, Indonesian, Bahasa Melayu) will be applied at the database level where platform functionality permits, and at the screening stage for databases that do not support language filtering.
