# Supplementary material for: Challenges, Strategies, and Explanatory Mechanisms in Clinical Skills Remediation Programs in Undergraduate and Postgraduate Medical Education in Low- and Middle-Income Countries: Realist Review Protocol
Source: JMIR Res Protoc. 2026 Jun 2;15:e89550. doi: 10.2196/89550 (PMC13229394; doi:10.2196/89550)
Supplement: Multimedia Appendix 3 [file resprot-v15-e89550-s003.docx]

**Protocol Title:** Challenges, Strategies, and Explanatory Mechanisms in Clinical Skills Remediation Programs in Undergraduate and Postgraduate Medical Education in Low- and Middle-Income Countries: A Realist Review Protocol

**PROSPERO Registration:** CRD42023447029

Version: 1.0 (Piloted on 5 training papers; duplicate extraction workflow aligned; April 2026)

Stage 1 design-control note: Each included source or document family will be assigned a Study Family ID and an evidentiary role (theory seeding, context mapping, theory testing, or administrative/background only). This is intended to reduce the researcher degrees of freedom and to distinguish documents that generate candidate mechanisms from those that confirm or challenge CMOCs.

**Instructions for Use:** This form is to be completed for each included study. Fields marked **(R)** are required for all studies. Fields marked **(O)** are optional/applicable where reported. Complete all sections before proceeding to CMO configuration extraction (Section G). Extraction will be conducted in NVivo for qualitative coding; document characteristics will be recorded in Microsoft Excel.

## SECTION A: Document Identification and Characteristics (R)

| Field | Entry |
| --- | --- |
| **A1. Unique Study ID** | [Assigned by primary extractor, e.g., CSRR-001] |
| **A2. Primary extractor** | [Initials] |
| **A3. Date of extraction** | [DD/MM/YYYY] |
| **A4. Secondary extractor (if applicable)** | [Initials] |
| **A5. Date of secondary extraction** | [DD/MM/YYYY] |
| **A6. First author surname** |  |
| **A7. Year of publication** |  |
| **A8. Full citation (APA 7th)** |  |
| **A9. DOI or URL** |  |
| **A10. Publication type** | ☐ Journal article ☐ Conference paper ☐ Thesis/dissertation ☐ Book chapter ☐ Report/policy document ☐ Grey literature ☐ Other: _______ |
| **A11. Language of publication** | ☐ English ☐ Indonesian ☐ Bahasa Melayu ☐ Other: _______ |
| **A12. Country of study** |  |
| **A13. World Bank income classification of study country (FY2026)** | ☐ Low-income ☐ Lower-middle-income ☐ Upper-middle-income ☐ High-income ☐ Multi-country (specify): _______ |
| **A14. LMIC classification** | ☐ LMIC ☐ HIC ☐ Mixed LMIC/HIC |
| **A15. Study design** | ☐ Randomised controlled trial ☐ Non-randomised controlled trial ☐ Cohort study (prospective) ☐ Cohort study (retrospective) ☐ Case-control study ☐ Cross-sectional study ☐ Case study/case report ☐ Qualitative study ☐ Mixed-methods study ☐ Programme evaluation ☐ Survey ☐ Theoretical/conceptual paper ☐ Systematic review ☐ Narrative review ☐ Grey literature ☐ Other: _______ |
| **A16. Funding source (if reported)** |  |
| **A17. Conflicts of interest declared?** | ☐ Yes ☐ No ☐ Not reported |
| A18. Study Family ID (if linked reports) | [Assigned when multiple reports describe the same programme/cohort] |
| A19. Evidentiary role in synthesis | ☐ Theory seeding ☐ Context mapping ☐ Theory testing ☐ Administrative/background only |

## SECTION B: Population Characteristics (R)

| Field | Entry |
| --- | --- |
| B1. Training level of participants | ☐ Undergraduate (preclinical) ☐ Undergraduate (clinical) ☐ Postgraduate (resident/registrar) ☐ Mixed undergraduate/postgraduate ☐ Other: _______ |
| B2. Medical specialty/discipline (if applicable) |  |
| B3. Year of training (if reported) |  |
| B4. Sample size (total) |  |
| B5. Sample size (remediation group, if applicable) |  |
| B6. Gender distribution (if reported) | % Male: ___ % Female: ___ % Not reported: ___ |
| B7. Age range or mean age (if reported) |  |
| B8. Prior academic performance (if reported) |  |
| B9. Inclusion of other health professions learners? | ☐ No ☐ Yes — specify: _______ |
| B10. Notes on population characteristics |  |

## SECTION C: Setting and Context (R)

| Field | Entry |
| --- | --- |
| C1. Healthcare setting | ☐ Hospital (secondary/tertiary care) ☐ Primary care/general practice ☐ Simulation centre ☐ Community setting ☐ Mixed settings ☐ Not specified |
| C2. Institutional type | ☐ University hospital ☐ Teaching hospital ☐ District/regional hospital ☐ Community health centre ☐ Not specified |
| C3. Resource context | ☐ Well-resourced (simulation, faculty, assessment infrastructure available) ☐ Moderately resourced ☐ Resource-constrained ☐ Not described |
| C4. Faculty capacity for remediation (if described) | ☐ Dedicated remediation faculty/staff ☐ General clinical supervisors ☐ Peer-assisted ☐ Not described |
| C5. Assessment infrastructure (if described) | ☐ Formal OSCE/structured assessment ☐ Direct observation ☐ Portfolio assessment ☐ Supervisor rating ☐ Multiple tools ☐ Not described |
| C6. Institutional framing of remediation | ☐ Developmental/supportive ☐ Disciplinary/punitive ☐ Mixed/unclear ☐ Not described |
| C7. Sociocultural context (if described) |  |
| C8. COVID-19 context (if applicable) | ☐ Pre-COVID (before 2020) ☐ During COVID disruption (2020–2022) ☐ Post-COVID (2022 onwards) ☐ Not applicable/not reported |
| C9. Notes on setting and context |  |

## SECTION D: Intervention Characteristics (R)

| Field | Entry |
| --- | --- |
| D1. Type of remediation intervention | ☐ Simulation-based ☐ Supervised clinical practice ☐ Peer-assisted learning ☐ Coaching/mentorship ☐ Structured feedback programme ☐ Structured reflection programme ☐ Multi-component programme ☐ Curriculum modification ☐ Online/remote remediation ☐ Other: _______ |
| D2. Explicit remediation context confirmed? | ☐ Yes (structured response to identified underperformance) ☐ No (general skills training only) ☐ Unclear |
| D3. Duration of remediation intervention |  |
| D4. Intensity (contact hours, sessions) |  |
| D5. Personnel delivering remediation | ☐ Faculty/academic staff ☐ Clinical supervisor ☐ Dedicated coach/mentor ☐ Peer ☐ Standardised patient ☐ Combination ☐ Not reported |
| D6. Assessment tools used to identify underperformance |  |
| D7. Assessment tools used to evaluate remediation outcomes |  |
| D8. Standalone or embedded in curriculum? | ☐ Standalone ☐ Embedded in curriculum ☐ Both ☐ Not reported |
| D9. Theory or framework underpinning the intervention (if stated) |  |
| D10. Notes on intervention characteristics |  |

## SECTION E: Outcome Data (R)

| Field | Entry |
| --- | --- |
| E1. Primary outcome(s) reported | ☐ Clinical skills competence score ☐ OSCE score ☐ Direct observation rating ☐ Supervisor rating ☐ Portfolio assessment ☐ Remediation completion rate ☐ Progression to next training stage ☐ Patient-safety outcome ☐ Other: _______ |
| E2. Competence outcome direction | ☐ Improvement ☐ No change ☐ Deterioration ☐ Mixed ☐ Not reported |
| E3. Competence outcome data (quantitative, if reported) | Pre-intervention: ___ Post-intervention: ___ Follow-up: ___ |
| E4. Remediation completion rate (if reported) |  |
| E5. Progression outcome (if reported) |  |
| E6. Patient-safety outcomes (if reported) |  |
| E7. Learner wellbeing outcomes (if reported) |  |
| E8. Professional identity outcomes (if reported) |  |
| E9. Faculty/resource outcomes (if reported) |  |
| E10. Unintended consequences (if reported) |  |
| E11. Follow-up period (if reported) |  |
| E12. Notes on outcomes |  |

## SECTION F: Mechanism and Process Data (R)

| Field | Entry |
| --- | --- |
| F1. Facilitators of remediation reported |  |
| F2. Barriers to remediation reported |  |
| F3. Learner psychological safety (if described) | ☐ Present — enabling ☐ Absent — inhibiting ☐ Mixed ☐ Not reported |
| F4. Feedback credibility (if described) | ☐ Perceived as credible ☐ Perceived as not credible ☐ Mixed ☐ Not reported |
| F5. Supervisory relationship quality (if described) | ☐ Trusting/supportive ☐ Threatening/evaluative ☐ Neutral ☐ Not reported |
| F6. Guided reflection component | ☐ Present ☐ Absent ☐ Not reported |
| F7. Professional identity impact (if described) | ☐ Identity threatened ☐ Identity reconstructed/restored ☐ Not reported |
| F8. Learner motivation (if described) | ☐ Intrinsically motivated ☐ Extrinsically motivated ☐ Resistant ☐ Not reported |
| F9. Perceived agency/control (if described) | ☐ High ☐ Low ☐ Not reported |
| F10. Hierarchical dynamics (if described) |  |
| F11. Sociocultural framing of remediation (if described) |  |
| F12. Notes on mechanisms and processes |  |

## SECTION G: CMO Configuration Extraction (R)

*This section is the core realist data extraction component. For each CMO configuration identified in the study, complete a separate row.*

### CMO Configuration Table

| CMO # | Context (C) — Conditions under which the mechanism is triggered | Mechanism (M) — The reasoning/response of participants that generates the outcome | Outcome (O) — The result generated by the mechanism in this context | Evidence source (quote/paraphrase + page/line number) | Theory contribution (supports / challenges / extends initial programme theory) | Domain alignment (Psychological Safety / Feedback Credibility / Guided Reflection / Identity Reconstruction / Hierarchy / Agency / Resource conditions / Other) |
| --- | --- | --- | --- | --- | --- | --- |
| CMO-1 |  |  |  |  |  |  |
| CMO-2 |  |  |  |  |  |  |
| CMO-3 |  |  |  |  |  |  |
| CMO-4 |  |  |  |  |  |  |
| CMO-5 |  |  |  |  |  |  |
| *(Add rows as needed)* |  |  |  |  |  |  |

**G1. Total number of CMO configurations extracted from this study:** ___

G2. Overall theory contribution of this study: ☐ Strongly supports initial programme theory ☐ Partially supports initial programme theory ☐ Challenges initial programme theory — specify: _______ ☐ Extends initial programme theory — specify: _______ ☐ Provides LMIC-specific contextual data ☐ Provides HIC data relevant to theory building ☐ Highlights cross-cutting influences of hierarchy, agency, or resource conditions

**G3. Key quotes supporting CMO configurations (copy verbatim with page/line numbers):**

## SECTION H: Rigour Assessment (R)

Data-fragment use rule: fragments judged rich in context but thin on mechanism may be used for context mapping only; fragments judged rich in mechanism but weakly linked to outcomes may be used for theory refinement but not CMOC confirmation; unsupported assertions will not be used as confirmatory evidence.

Complete the appropriate rigour appraisal tool based on the study design below:

| Field | Entry |
| --- | --- |
| H1. Rigour appraisal tool applied | ☐ RoB 2 (randomised controlled trials) ☐ ROBINS-I (experimental/quasi-experimental) ☐ CASP Qualitative Checklist ☐ MMAT (mixed-methods) ☐ Source credibility assessment (grey literature) |
| H2. Overall rigour rating | ☐ Low risk of bias / High rigour ☐ Moderate risk of bias / Moderate rigour ☐ High risk of bias / Low rigour ☐ Critical limitations |
| H3. Rigour assessment notes |  |
| H4. Decision for inclusion | ☐ Include — high relevance and sufficient rigour ☐ Include with caveats — moderate rigour but unique theoretical/contextual contribution ☐ Exclude — critical rigour limitations that undermine theory contribution ☐ Refer to third reviewer (YS) |
| H5. Reason for inclusion/exclusion |  |

## SECTION I: Extractor Notes and Queries (O)

| Field | Entry |
| --- | --- |
| **I1. Queries for team discussion** |  |
| **I2. Potential links to other included studies** |  |
| **I3. Supplementary searches triggered by this study** |  |
| **I4. Any translation issues (for Indonesian/Bahasa Melayu sources)** |  |

## Calibration Exercise Protocol

Prior to formal data extraction, all extractors will complete this form independently for **five training papers** selected to represent the range of study designs and LMIC/HIC contexts expected in the full review. Inter-rater reliability will be assessed using Cohen’s kappa for categorical items and percentage agreement for text items. A kappa of ≥0.70 will be considered acceptable. Systematic discrepancies identified during calibration will be resolved through discussion and protocol clarification before full extraction proceeds.
